# Identification of novel DNA methylation inhibitors via a two-component reporter gene system

**DOI:** 10.1186/1423-0127-18-3

**Published:** 2011-01-10

**Authors:** Yi-Shiuan Lin, Arthur Y Shaw, Shi-Gang Wang, Chia-Chen Hsu, I-Wen Teng, Min-Jen Tseng, Tim HM Huang, Ching-Shih Chen, Yu-Wei Leu, Shu-Huei Hsiao

**Affiliations:** 1 Human Epigenomics Center, Department of Life Science, Institute of Molecular Biology and Institute of Biomedical Science, National Chung Cheng University, Chia-Yi, 621, Taiwan; 2 Department of Pharmacology and Toxicology, College of Pharmacy, Southwest Comprehensive Center for Drug Discovery and Development, University of Arizona, Tucson, AZ 85721, USA; 3 Human Cancer Genetics Program, Department of Molecular Virology, Immunology, and Medical Genetics, and the Comprehensive Cancer Center, The Ohio State University, Columbus, OH 43210, USA; 4 Division of Medicinal Chemistry, College of Pharmacy, The Ohio State University, Columbus, OH 43210, USA

## Abstract

**Background:**

Targeting abnormal DNA methylation represents a therapeutically relevant strategy for cancer treatment as demonstrated by the US Food and Drug Administration approval of the DNA methyltransferase inhibitors azacytidine and 5-aza-2'-deoxycytidine for the treatment of myelodysplastic syndromes. But their use is associated with increased incidences of bone marrow suppression. Alternatively, procainamide has emerged as a potential DNA demethylating agent for clinical translation. While procainamide is much safer than 5-aza-2'-deoxycytidine, it requires high concentrations to be effective in DNA demethylation in suppressing cancer cell growth. Thus, our laboratories have embarked on the pharmacological exploitation of procainamide to develop potent DNA methylation inhibitors through lead optimization.

**Methods:**

We report the use of a DNA methylation two-component enhanced green fluorescent protein reporter system as a screening platform to identify novel DNA methylation inhibitors from a compound library containing procainamide derivatives.

**Results:**

A lead agent IM25, which exhibits substantially higher potency in *GSTp1 *DNA demethylation with lower cytotoxicity in MCF7 cells relative to procainamide and 5-aza-2'-deoxycytidine, was identified by the screening platform.

**Conclusions:**

Our data provide a proof-of-concept that procainamide could be pharmacologically exploited to develop novel DNA methylation inhibitors, of which the translational potential in cancer therapy/prevention is currently under investigation.

## Background

As DNA methylation-mediated silencing of genes has been implicated in the pathogenesis of many diseases including cancer [1-7], targeting aberrant DNA methylation is considered as a therapeutically relevant strategy for cancer treatment. Among many agents with DNA methylation-modifying capability, 5-aza-2'-deoxycytidine (decitabine; 5-Aza) is the best-known DNA demethylation agent. 5-Aza exerts its effect by inhibiting DNA methyltransferases (DNMTs), the key enzymes responsible for initiating or maintaining the DNA methylation status, thereby facilitating the re-expression of tumor suppressor genes through DNA hypomethylation. Its therapeutic efficacy is manifest by the Food and Drug Administration approval for the treatment of myelodysplastic syndromes. While 5-Aza is a potent DNA demethylation agent [8,9], its use is associated with increased incidences of bone marrow suppression, including neutropenia and thrombocytopenia, due to the disruption of DNA synthesis. In addition, shorter half-life hinders the effective delivery of 5-Aza to the tumor site [10].

Recently, procainamide has emerged as a potential DNA demethylating agent for clinical translation. Evidence indicates that procainamide inhibits DNMT1 by reducing the affinity with its two substrates: hemimethylated DNA and *S*-adenosylmethionine [11-13]. Through DNA demethylation, procainamide causes growth arrest [9] and reactivation of tumor suppressor genes in cancer cells [14]. Moreover, as an anti-arrhythmic drug, procainamide has a well-characterized safety profile without side effects commonly associated with nucleoside analogues [15,16]. However, in contrast to 5-Aza, procainamide requires high concentrations (≥ 50 μM) to be effective in DNA demethylation in suppressing cancer cell growth [9,11]. Thus, our laboratories have embarked on the pharmacological exploitation of procainamide to develop potent DNA methylation inhibitors through lead optimization.

Previously, we reported a two-component enhanced green fluorescent protein (EGFP) reporter gene system for the visualization and quantization of dynamic changes in targeted DNA methylation in bone marrow-derived mesenchymal stem cells or cancer cell lines [17,18]. This system gives a direct and concomitant measurement and evaluation of DNA demethylation and cytotoxicity in living cells, thus providing an expedient screening platform for identifying demethylating agents. As the exact mode of action of procainamide in decreasing the binding DNMT1 with its substrate remains undefined, we used procainamide as a scaffold to develop a focused compound library, which in combination with other in-house compound libraries, was used for screening via this two-component system.

## Methods

### Cell culture and drug treatment

MCF7 breast cancer cells, obtained from American Type Culture Collection, were grown in Minimal Essential Medium (MEM; Invitrogen), supplemented with 10% FBS, 2 mM L-glutamine, and 100 μg/ml penicillin/streptomycin. Cells were cultured at 37°C in a humidified incubator containing 5% CO_2_. Medium changes were performed twice weekly and cell passages were performed at 90% confluence. To maintain the two-component constructs in MCF7 cells, 200 μg/mL of hygromycin B (Invitrogen) and 500 μg/mL of Geneticin (G418, Calbiochem) were included in culture medium. 5-Aza and procainamide were purchased from Sigma-Aldrich. Synthesis of procainamide derivatives and other tested agents (structures, Additional file 1: Figure S1) will be described elsewhere. Tested agents were dissolved in DMSO as stock solutions, and added to culture medium with final DMSO concentrations of 0.3% and 1.2% (v/v) for 7.5 μM and 30 μM of testing drugs, respectively. Control cells received DMSO vehicle. During the 5-day treatment period, medium was changed on the third day of treatment along with the addition of 17β-estrodial (E2, 10 ng/ml).

### *In vitro *DNA methylation

PCR-amplified and purified *Trip10 *promoter (4 μg) was incubated with 20 U of CpG methyltransferase (*Sss*I, New England BioLabs) at 37°C for 4 h in the presence of 160 μM *S*-adenosylmethionine to induce methylation at the *Trip10 *promoter DNA. Complete conversion was indicated by the resistance of methylated *Trip10 *DNA to methylation-sensitive restriction enzymes (*Hpa*II, New England BioLabs).

### Transfection

*In vitro *methylated *Trip10 *promoter DNAs (0.4 μg) were denatured and used to transfect 1 × 10^5 ^cells/well in 6-well plate at day 1, 3, and 5 using DMRIE-C (Invitrogen) according to the manufacturer's instruction. Unmethylated PCR products were transfected as mock controls. Tracking of the transfected DNAs was performed by using the *Label*IT Tracker Cy5 Intracellular Nucleic Acid Localization Kit (Mirus) by following the manufacture's instruction. Cells were monitored by fluorescence microscopy (Axiovert 200M, ZEISS) and analyzed with the MetaMorph software.

### Bisulfite conversion

Genomic DNA (0.5 μg) was bisulfite-converted by the EZ DNA Methylation Kit (Zymo) by following the manufacturer's instruction.

### Qmsp

Bisulfite converted DNAs were PCR-amplified by using PCR primers listed in Additional file 1: Table S1. Universal methylated DNAs (Millipore) were used as positive control. *Col2A1 *was used as a loading control and to amplify the serial diluted (1/10, 1/100 and 1/1000) bisulfite-converted universal methylated DNAs to generate the standard curve for quantization in real time PCR machine (Bio-Rad, iQ5). The methylation percentage was calculated as [(Intensity of Amplifications by *Trip10 *MSP primer set) × 100]/(Intensity of Amplifications by *Col2A1 *MSP primer set) (%). The qMSP was performed in a 25 μL of reaction mixture containing 4 μL of template (bisulfite treated DNA), 2 μL of primer pair (2.5 μM), 12.5 μL of 2X reaction buffer (SYBR Green real time PCR Master Mix, Toyobo), and 6.5 μL of H_2_O.

**Analysis of EGFP expression **was performed by fluorescence microscopy (Axiovert 200M, ZEISS) with the MetaMorph software. Images and intensities of EGFP of more than 600 cells were analyzed by the image analysis program NIH Image (Image J).

### Western blotting

Equal amounts of protein in cell lysates were separated in 10% SDS polyacrylamide gels and then transblotted to nitrocellulose membranes. After blocking with non-fat milk, the membrane was washed and incubated with antibodies against GFP (Abcam) or β-actin (Sigma-Aldrich). The membrane was washed, and then incubated with anti-rabbit immunoglobulin G-horseradish peroxidase. After final wash, the proteins were visualized by enhanced chemiluminescence. Immunoblotting data were analyzed by NIH Image.

### Cell viability assay

Cell viability was assessed using MTT [3-(4,5-dimethylthiazol- 2-yl)-2,5-diphenyl-2H-tetrazolium bromide, Sigma-Aldrich) in six replicates. MCF7 cells were seeded at 4,000 cells per well in 96-well plates, and treated with test agents at different concentrations. After 5-day treatment, cells were incubated in medium containing 0.5 mg/ml MTT at 37°C for 4 h. Reduced MTT was solubilized in 200 μl/well of DMSO for determination of absorbance of 595 nm using a microplate reader.

### ELISA

Enzyme-linked immunosorbent assay (ELISA) was performed using the GFP ELISA kit (Cell Biolabs, San Diego, CA) according to the manufacture's instruction.

### Differential Methylation Hybridization (DMH) Array

DMH was performed according to a reported procedure [19]. Amplicons prepared from control (vehicle-treated) MCF7 cells were labeled with Cy5 and the amplicons prepared from drug-treated cells were labeled with Cy3. Labeled DNAs were co-hybridized onto 244K Agilent promoter array. Array hybridization results were normalized with LOWESS and a cutoff of 4 folds was used.

### Statistics

ELISA and MTT results were analyzed by *F*-test. Significant demethylated loci were confirmed by ANOVA from DMH results. Linear regression was used to deduce the global methylation states before and after the demethylation agent treatment.

## Results

### Monitoring DNA methylation status by a two-component EGFP reporter gene system

**Figure 1:**
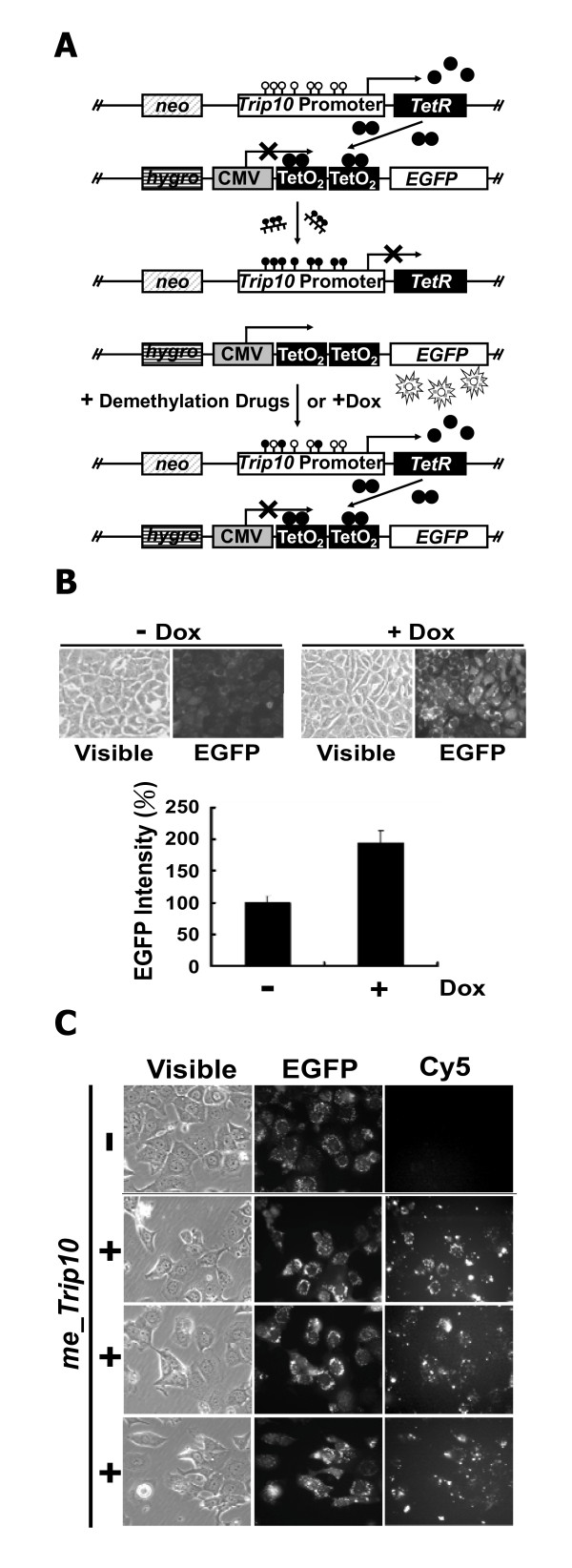
**Screening system for demethylation agents**. (A) Schematic depiction of the reporter system in MCF7 cells. The reporter gene system consists of two constructs (top). Expression of Tet repressor (*TetR*) was under the regulation of *Trip10 *promoter. The addition of doxycycline (Dox) or targeted methylation of *Trip10 *promoter (center) blocks the silencing/binding of Tet repressor onto the Tet operator (TetO_2_) thus the EGFP would express and become detectable with fluorescence microscope. Bottom, demethylation of the *Trip10 *promoter restores the expression of Tet repressor and thus silences the EGFP. Open circles: unmethylated CpG dinucleotides; filled circles: methylated CpG dinucleotides. (B) Validation of the reporter gene system. As shown, presence of doxycycline did not affect the cell density, but increased the intensity of EGFP substantially (top). Histograms show the quantified EGFP intensity in the presence or absence of doxycycline (bottom). The EGFP intensity was analyzed and quantified by Image J. Columns, mean (n = 3); error bars, S.D. (C) Tracking of the transfected *me_Trip10 *DNAs. *In vitro *methylated *Trip10 *promoter DNAs were labelled with or without (control) Cy5 and then used to transfect the MCF7 cells. There is a high degree of overlap between the EGFP-expressing cells and Cy5-positive cells, suggesting the high transfection efficiency in this system.

We previously reported the development of a two-component EGFP reporter gene system to confirm the level of transcriptional silencing and to visualize DNA methylation of the *Trip10 *promoter in the differentiation of bone marrow-derived mesenchymal stem cells [17]. In this study, we further applied this DNA methylation-targeted reporter system for the screening of compound libraries to identify novel DNA methylation inhibitors. As illustrated in Figure 1A, the system consists of two constructs. The first construct consists of the CpG-rich *Trip10 *promoter region, which governs the expression of downstream *Tet *repressor. The *Trip10 *promoter also contains an estrogen receptor (ER)-binding site, which is essential for the gene expression in ER-positive MCF7 breast cancer cells. The second construct contains two Tet repressor-binding sites, *Tet o*perator (*TetO*_*2*_), which is located between the viral *CMV *promoter and the *EGFP *reporter gene. When the *Trip10 *promoter in the first construct was un-methylated, the expressed Tet repressor would block the EGFP expression by binding to the *TetO*_*2*_. Conversely, methylation of *Trip10 *promoter would suppress the expression of Tet repressor and thus de-repress the EGFP expression. These two constructs were co-transfected into MCF7 cells, and transfectants containing both constructs were selected by hygromycin and G418 and confirmed by PCR. To validate the co-transfection and the two-component regulation, doxycycline (Dox) was added to interfere with the expressed Tet repressor and de-repress the EGFP expression (Figure 1B). To examine the transfection efficiency, the *in vitro *methylated *Trip10 *(*me_Trip10*) DNAs were coupled with Cy5 and then transfected into the MCF7 cells. Our data indicate a strong correlation between Cy5-expression and EGFP expression in these cells (Figure 1C), suggesting high transfection efficiency.

**Figure 2:**
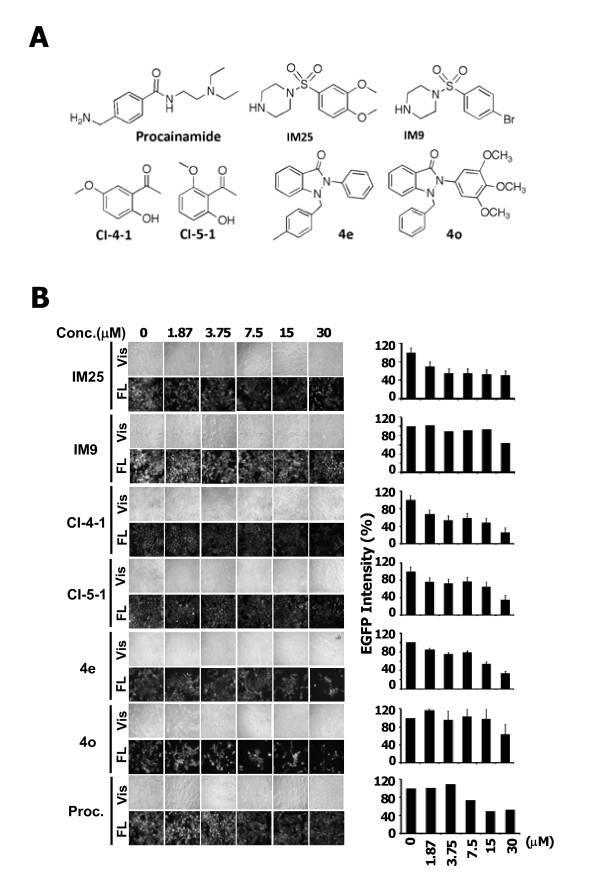
**Identification of optimal DNA demethylating agents**. (A) Chemical structures of procainamide, the three selected agents IM25, CI-4-1, and 4e and their structurally related analogues IM9, CI-5-1, and 4o. (B) Potencies of procainamide, IM25, IM9, C-4-1, CI-5-1, 4e, and 4o in attenuating EGFP expression in the two-component reporter system. The EGFP intensities in individual treatments are illustrated on the right. Columns, mean (n = 3); error bars, S.D.

**Figure 3:**
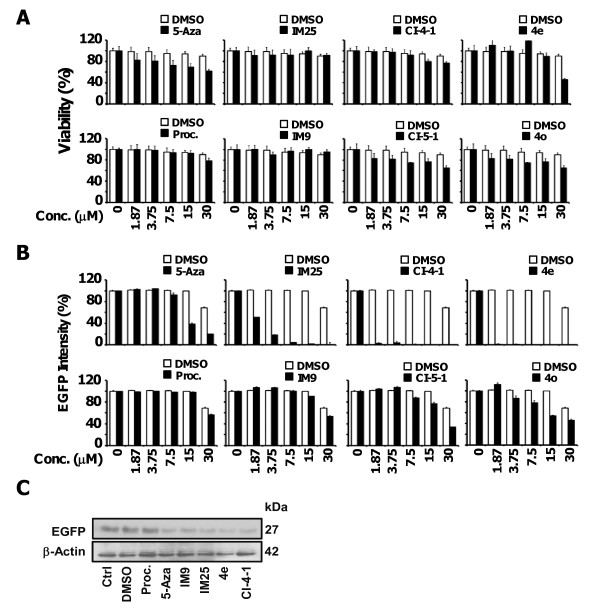
**Potency and safety of DNA demethylating agents**. (A) Comparison of the dose-dependent suppressive effects of 5-Aza, procainamide, IM25, and IM9 on the viability of MCF7 cells by MTT assays. Columns, mean (n = 3); error bars, S.D. (B) Parallel ELISA analysis of the suppressive effects on EGFP expression in the two-component reporter system. Columns, mean (n = 3); error bars, S.D. (C) Western blot analysis of the effects of procainamide, 5-Aza, IM9, and IM25, each at 7.5 μM, on EGFP expression.

We further validated this two-component EGFP reporter gene system by exposing the transfected MCF7 cells to different concentrations of 5-Aza and procainamide. After 5-day treatment, both agents showed dose-dependent, repressive effects on EGFP expression levels relative to DMSO control, as visualized by fluorescence microscopy, with relative potency of 5-Aza greater than procainamide (Additional file 1: Figure S2 and Figure 2B). Equally important, as these two drugs did not cause morphological changes [light microscopy (Vis), Additional file 1: Figure S2] or suppression of cell viability (Figure 3A, Statistics in Additional file 1: Table S2), these findings indicate that this reduction in EGFP expression was not caused by drug-induced cell death, suggesting a direct consequence of DNA demethylation.

### Screening of a compound library using the two-component reporter system

After validating this reporter system, we set to assess the abilities of individual compounds in our compound library, each at 7.5 μM, to mediate DNA demethylation. This library consisted of 169 compounds, the majority of which were procainamide derivatives (Additional file 1: Figure S1). Based on this screening platform, 46 derivatives significantly inhibited EGFP expression, of which 36 did not cause apparent cell death (Additional file 1: Figure S3). Interestingly, these 36 compounds belonged to three structural series, i.e., IM, CI, and 4x, of which IM25, CI-4-1, and 4e (structures, Figure 2A) represented optimal agents. The dose-responses of these agents, along with three structurally related analogues IM9, CI-5-1 and 4o, in suppressing EGFP expression are depicted in Figure 2B.

These agents vis-à-vis 5-Aza and procainamide were further subjected to ELISA (statistics in Additional file 1: Table S3) and Western blotting to assess their abilities to suppress EGFP expression as well as MTT assays (statistics in Additional file 1: Table S2) for their respective cytotoxicity. As revealed in Figure 3A and, only 5-Aza, 4e, and 4o at high concentrations (30 μM) substantially suppressed MCF7 cell viability, while other compounds examined had no apparent effect (statistic in Additional file 1: Table S2). Data obtained from ELISA assay support the visualization results in Figure 2B, in which IM25 exhibits greater demethylation potency than 5-Aza in terms of inhibiting the EGFP expression.

In another set of experiment, cells were treated with 7.5 μM of candidate drugs for 5 days, then the cell lysates were harvested and subjected to Western blot. As shown in Figure 3C, the protein level of EGFP was substantially suppressed by IM25 as well. However, we did notice that the protein level appears to be significantly inhibited by IM9 as well. The inconsistence between the Western blot results versus the ELISA/images is not immediately clear. Results obtained from ELISA and Western blot confirmed that IM25 is an effective demethylation agent in inhibiting EGFP expression.

### Targeted and global loci demethylation

**Figure 4:**
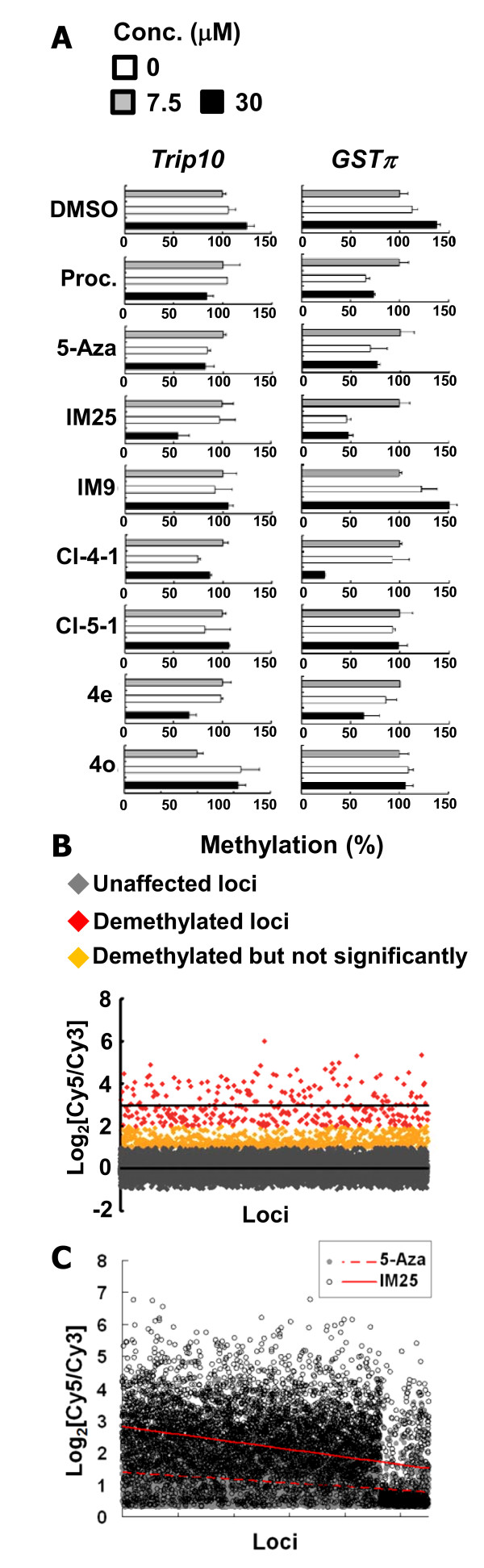
**Drug-induced loci-specific and global demethylation**. (A) MSP was applied to determine the methylation level of *Trip10 *and *GSTp1 *in MCF7 cells treated with each of the following derivatives at 7.5 or 30 μM: procainamide, 5-Aza, IM25, IM9, CI-4-1, CI-5-1, 4e, and 4o, after 5 days of treatment. Procainamide, 5-Aza, IM25, CI-4-1 and 4e significantly reduced the methylation level of *GSTp1*, while they appear to have weaker effect in demethylating *Trip10 *promoter (B) DMH analysis of the effect of 7.5 μM IM25 on genome-wide methylation. The higher Log_2_[Cy5/Cy3] values indicate stronger demethylation effects. (C) Genome-wide comparison of the demetylation after IM25 and 5-Aza treatment. DMH microarrays identified 5,506 more loci that are demethylated after IM25 treatment (open circles) than the result obtained after 5-Aza treatment (close gray circles). The regressed lines depicted the distribution of demethylation (dashed line for 5-Aza; solid line for IM25) and all the listed 5,506 loci were confirmed significant by ANOVA.

Both 5-Aza and procainamide have been shown to cause demethylation of *GSTp1 *(*GSTπ1*) in colon, prostate, and breast cancer cells [11-14]. Thus, we conducted MSP to determine the DNA methylation levels of *Trip10 *and *GSTp1 *in MCF7 cells treated with IM25, CI-4-1, and 4e vis-à-vis 5-Aza and procainamide. Among these testing compounds, IM25 exhibits the greatest potency in facilitating the demethylation of both *Trip10 *and *GSTp1*, CI-4-1 also decreases the methylation level of both genes, but to a lesser extent (Figure 4A). Compound 4e showed a slight demethylation of *GSTp1 *only at 30 μM. IM25 was chosen to further elucidate its genome-wide demethylation effects. Cells treated with either IM-25 or DMSO were harvested and subjected to DMH. As demonstrated in Figure 4B, IM25 was able to cause global demethylation in MCF7 cells. When comparing the global demethylation capacity between IM25 and 5-Aza, 5,506 more demethylated loci out of 244K target loci in Agilent array were identified (Figure 4C, significant loci confirmed by ANOVA). Therefore, the identified IM25 possesses equal or more demethylation capacity than the known drugs such as 5-Aza and procainamide.

## Discussion

Since the discovery that DNA methylation-mediated gene silencing is a reversible event, identifying small-molecule DNA methylation inhibitors, either natural or synthetic, for cancer treatment has been the focus of many investigation [10]. Although 5-Aza is the most potent demethylating agent in clinical use, general side effects associated with this nucleoside drug such as bone marrow suppression might be of concern. Thus, continuing efforts have been made to search for additional DNA methylation inhibitors with greater potency but low cytotoxicity. Procainamide, anti-arrhythmic drug, has emerged as a potential candidate because of its well-characterized safety profile. However, its clinical translation might be hindered by its relatively low potency in inhibiting DNA methylation. Thus, our laboratories have embarked on the pharmacological exploitation of procainamide to identify novel DNA demethylating agents through the unique screening platform we have developed.

In the present study, IM25, a procainamide derivative, is found to be as potent as 5-Aza in terms of demethylation but with much lower cytotoxicity. Azacytidine and 5-Aza have been approved for the treatment of myelodysplastic syndromes by FDA, and they are currently under clinical trials for treatment of solid tumors including breast cancer, lung cancer, and colon cancer [20-28]. On the other hand, accumulating data indicate that these demethylation agents could also control stem cell differentiation [29] and treat diseases like sickle cell disease [30]. Despite these promising clinical and *in vitro *studies for cancer treatment and cell fate manipulation, the side effects such as bone marrow suppression has always been a great concern. Our global demethylation analysis by DMH and single target gene validation by MSP indicate that IM25 treatment could disturb MCF7 cell's epigenome. However, IM25 did not cause overt cell death even at rather high concentrations. We reason that IM25 treatment may act via altering the gene expression profile and subsequently cause changes in cell physiology/behaviour without affecting cell viability. It will be crucial to determine if IM25 has the same demethylation potency and safety in other cancer cell types or stem cells. In addition, to determine the IM25' effect in an animal model system will provide in-depth information regarding its efficacy and safety upon systemic administration, which can serve as justification for its future clinical application as a direct antitumor drug, an adjuvant, or other therapeutic uses.

On the other hand, present study also demonstrates the feasibility of the established two-component reporter system as an efficient drug screening platform in live cells. While the efficacy of 5-Aza in reducing *GSTp1 *methylation is lower in the two-component system than in the MSP assay, which possibly is attributed to the engineered two-component stable clones, we reason that the direct, non-invasive measurement and comparisons of testing drugs in the two-component platform can serve as a powerful while economic tool for rapid drug screening at preclinical stage.

## Conclusions

In summary, the impetus of this study is twofold. First, our DNA methylation-targeted two-component reporter system represents an expedient screening platform to identify agents with DNA methylation-altering capability. Second, we have identified the procainamide derivative IM25 which exhibits high global demethylation potency, of which the translational value in cancer treatment represents the focus of this investigation.

## Competing interests

The authors declare that they have no competing interests.

## Authors' contributions

YSL, SGW, CCH, I.-W. Teng and MJT performed the experiments; AYS and CSC designed and synthesized the compound library; THMH, CSC, SHH and YWL supervised the research and wrote the manuscript. All authors read and approved the final manuscript.

## Supplementary Material

Additional file 1 **Supplementary materials**. Additional file contains the supplementary materials which include: Supplementary Figures S1 to S3 and Supplementary Tables S1 to S3 Click here for file
