# Supplementary material for: Identification of novel DNA methylation inhibitors via a two-component reporter gene system
Source: J Biomed Sci. 2011 Jan 10;18(1):3. doi: 10.1186/1423-0127-18-3 (PMC3025941; doi:10.1186/1423-0127-18-3)
Supplement: Additional file 1 — Supplementary materials. Additional file contains the supplementary materials which include: Supplementary Figures S1 to S3 and Supplementary Tables S1 to S3 [file 1423-0127-18-3-S1.PDF]

Identification of Novel DNA Methylation Inhibitors via a Two-Component Reporter Gene System

Yi-Shiuan Lin, Arthur Y. Shaw, Shi-Gang Wang, Chia-Chen Hsu, I-Wen Teng, Min-Jen Tseng, Tim H.-M. Huang, Ching-Shih Chen, Yu-Wei Leu and Shu-Huei Hsiao

This file includes:

Supplementary Figures S1 to S3

Supplementary Tables S1 to S3

Supplementary Figure Legends

Figure S1 Chemical structures of individual compounds of the compound library used for screening.

Figure S2 Visualization and quantification of the demethylation effects by 5-Aza, procainamide, and DMSO (vehicle). Various concentrations of 5-Aza and procainamide or equal volume of DMSO was added into the culture medium for 5 days. EGFP intensity is visibly decreased as a function of 5-Aza or procainamide concentrations.

Figure S3 Comparison of demethylation effects of the 169 compound library. These derivatives were used to treat the MCF7 cells with two-component reporter system with the same concentration (7.5 μM) for 5 days. Drugs that result in dimed EGFP intensity without apparent changes in cell viability were highlighted with green boxes, whereas those displayed cell death were highlighted with red boxes. A total of 36 derivatives were found to reduce EGFP intensity without causing cytotoxicity and thus considered to be potential demethylation candidates.

Supplementary Figure S1

Cpd	Structure	Cpd	Structure	Cpd	Structure	Cpd	Structure
SC-4 m-2	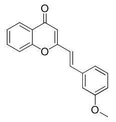	SC-4 m-9	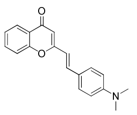	SC-4 m-16	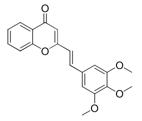	BNO-2	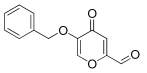
SC-4 m-3	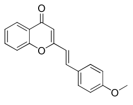	SC-4 m-10	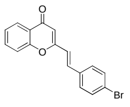	BAO-1	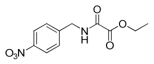	KOJ-1	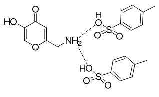
SC-4 m-4	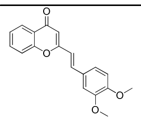	SC-4 m-11	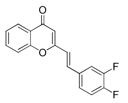	BAO-2	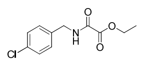	KOJ-2	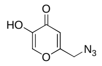
SC-4 m-5	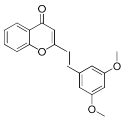	SC-4 m-12	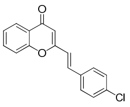	PMK-1	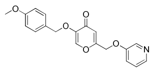	KAI-3	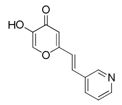
SC-4 m-6	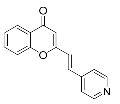	SC-4 m-13	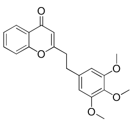	PMK-2	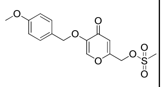	OBn-KAI-8	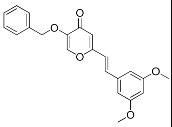
SC-4 m-7	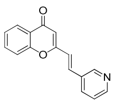	SC-4 m-14	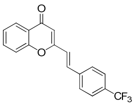	PMK-3	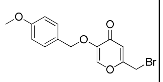	PMB-KAI-A	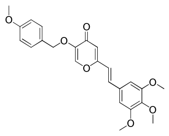
SC-4 m-8	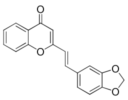	SC-4 m-15	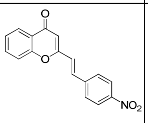	BNO-1	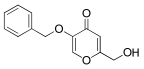	PMB-KAI-3	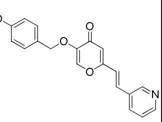

Supplementary Figure S1

**PMB-KAI-2** 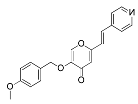	**SC-4g** 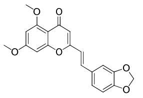	**SC-4o** 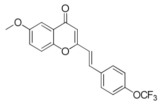	**CI-3-2** 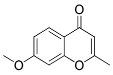
**KAI-1** 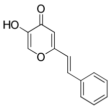	**SC-4h** 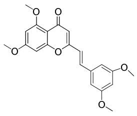	**SC-4p** 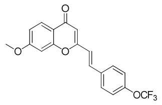	**CI-4-1** 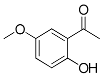
**SC-4a** 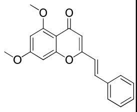	**SC-4i** 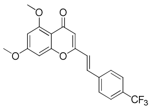	**SC-4a-2** 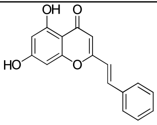	**CI-4-2** 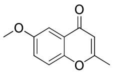
**SC-4b** 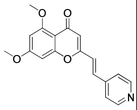	**SC-4j** 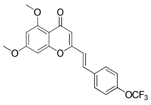	**SC-4f-2** 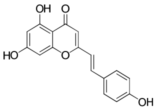	**CI-5-1** 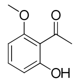
**SC-4c** 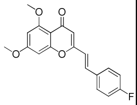	**SC-4k** 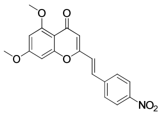	**SC-4j-2** 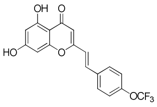	**CI-5-2** 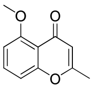
**SC-4d** 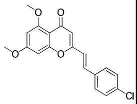	**SC-4l** 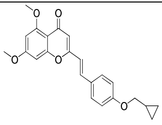	**CI-1-1** 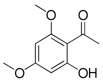	**CI-6-1** 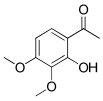
**SC-4e** 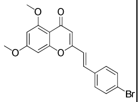	**SC-4m** 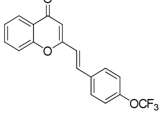	**CI-1-2** 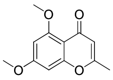	**CI-6-2** 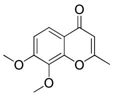

Supplementary Figure S1

M1	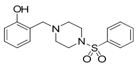	M16	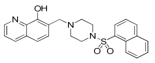	M34	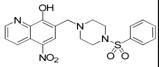	IM15	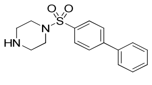
M2	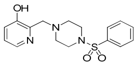	M17	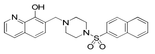	M35	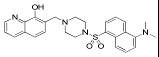	IM18	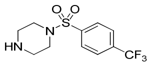
M5	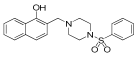	M18	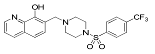	Q1	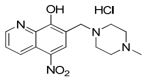	IM19	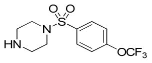
M6	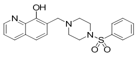	M19	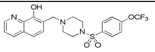	Q2	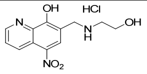	IM24	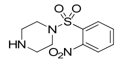
M7	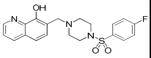	M22	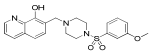	T-1	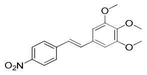	IM25	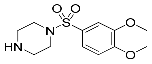
M8	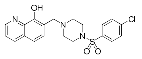	M24	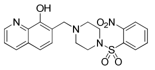	IM6	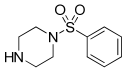	IM32	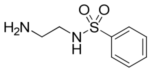
M9	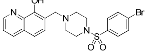	M25	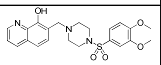	IM7	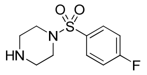	IM35	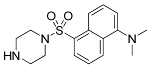
M10	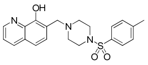	M26	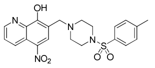	IM8	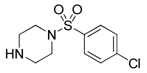	Kia-P-12	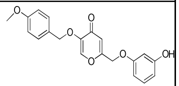

Supplementary Figure S1

M11	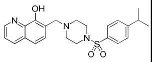	M28	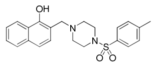	IM9	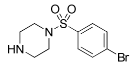	Kia-B-12	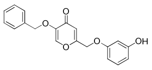
M12	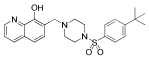	M29	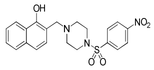	IM11	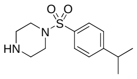	Kia-13	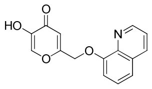
M13	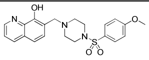	M32	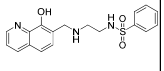	IM12	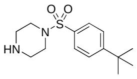	Kia-B-11	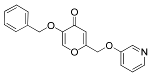
M15	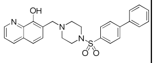	M33	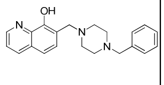	IM14	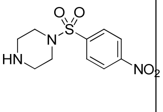	OXA-C	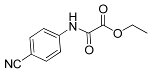
OXA-B	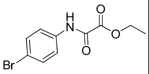	PY-4	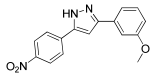	IM-1	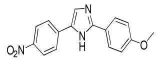	QN-5	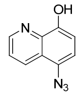
OXA-T	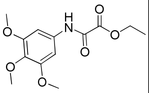	PY-5	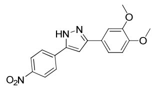	IM-2	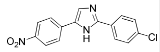	AA-1	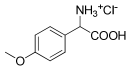
OXA-O	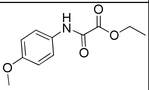	PY-7	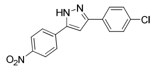	OXZ-1	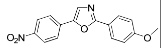	AA-2	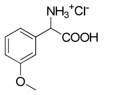
OXA-2	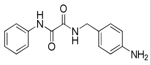	PY-11	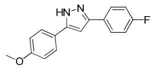	OXZ-2	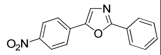	AA-3	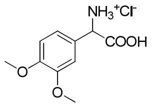
OXA-3	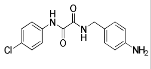	PY-13	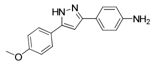	OXZ-3	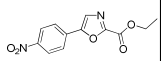	AA-4	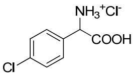

Supplementary Figure S1

OXA-5	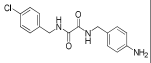	PY-14	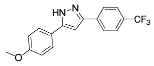	SC-4h-2	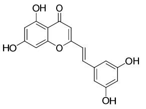	AA-5	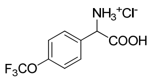
OXA-4	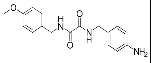	PY-15	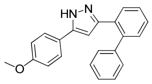	QN-1	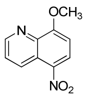	4b	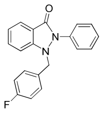
PY-1	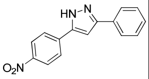	PY-16	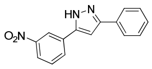	QN-2	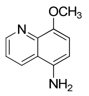	4c	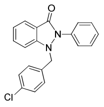
PY-2	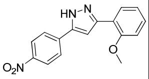	PY-17	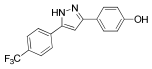	QN-3	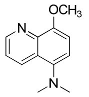	4d	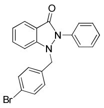
4f	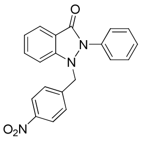	4n	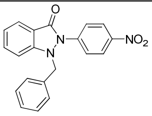	3b	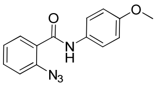	3f	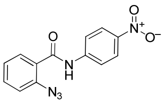
4g	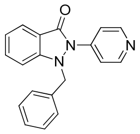	4o	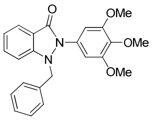	3c	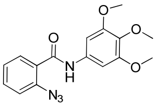	3g	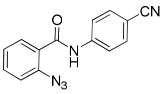
4i	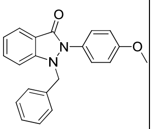	3i	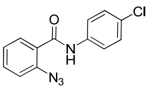	3d	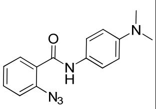	3h	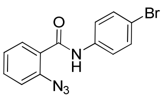
4k	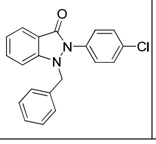	3a	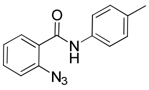	3e	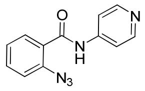		

Supplementary Figure S2

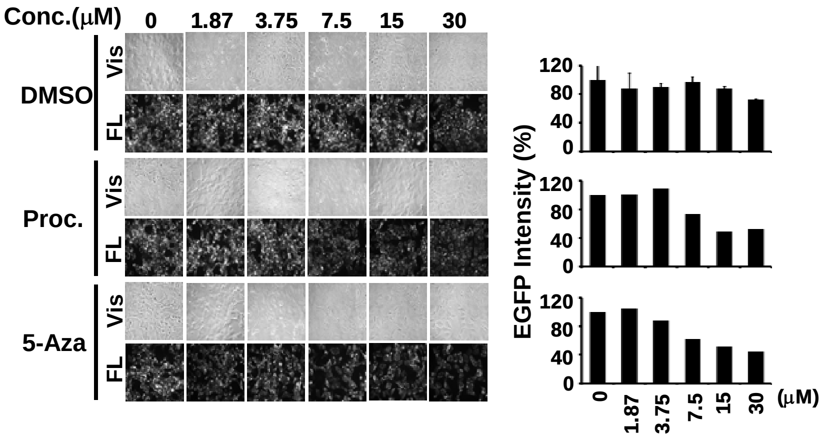

Supplementary Figure S3

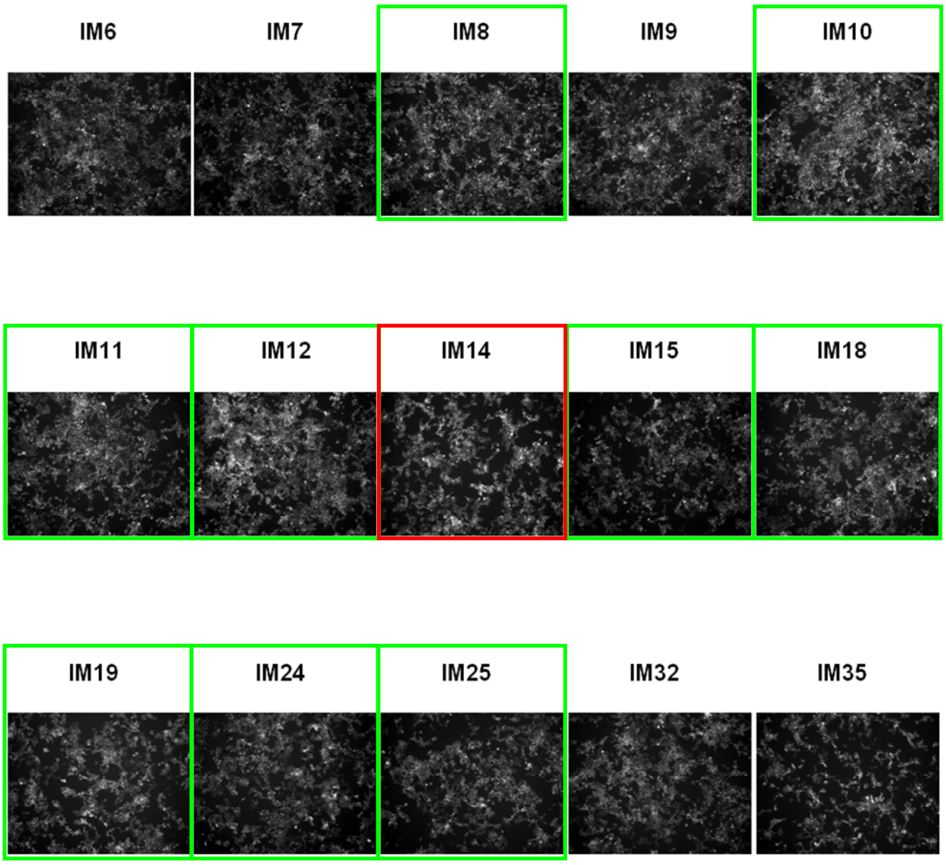

Supplementary Figure S3

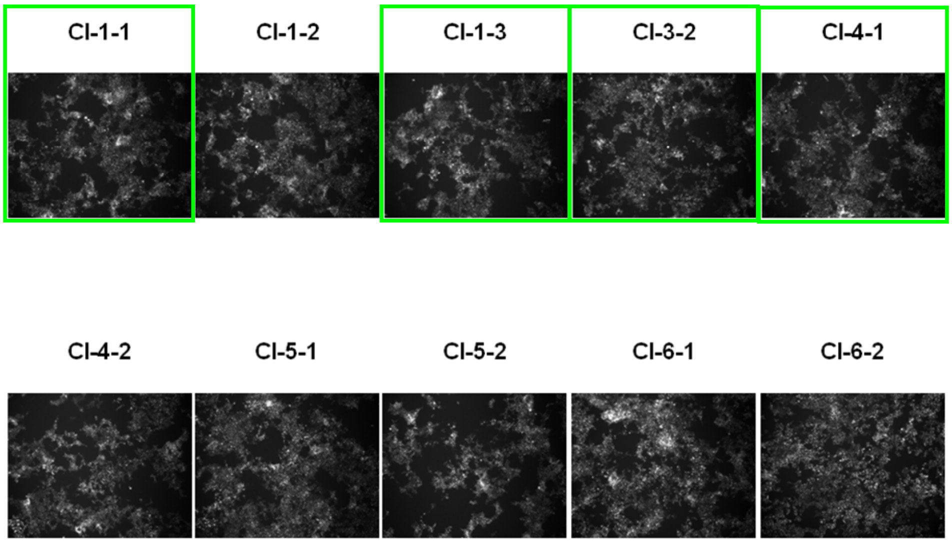

Supplementary Figure S3

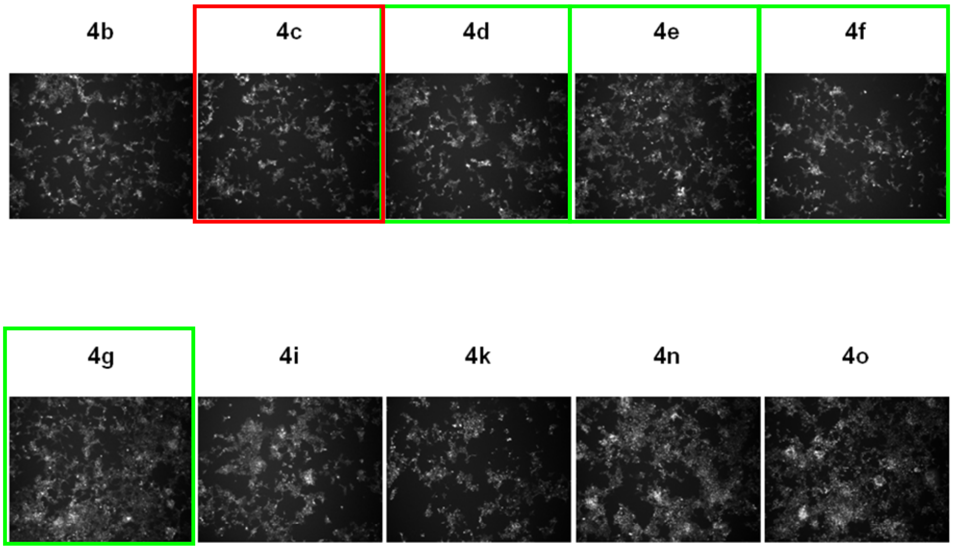

Supplementary Figure S3

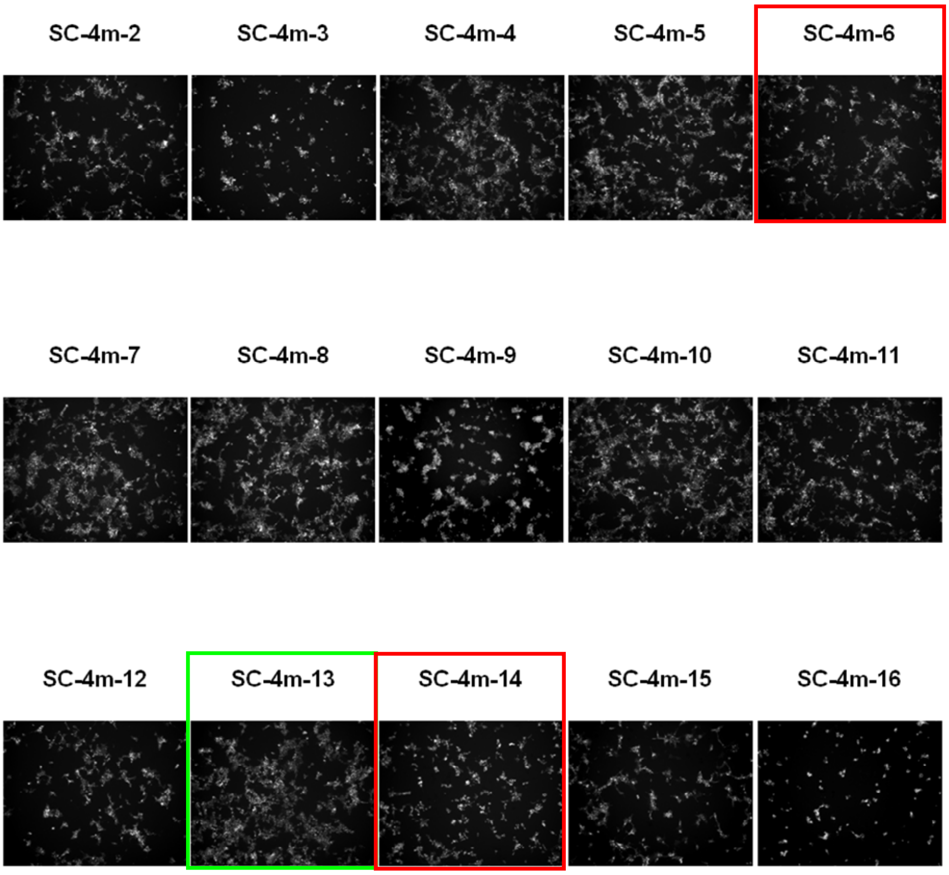

Supplementary Figure S3

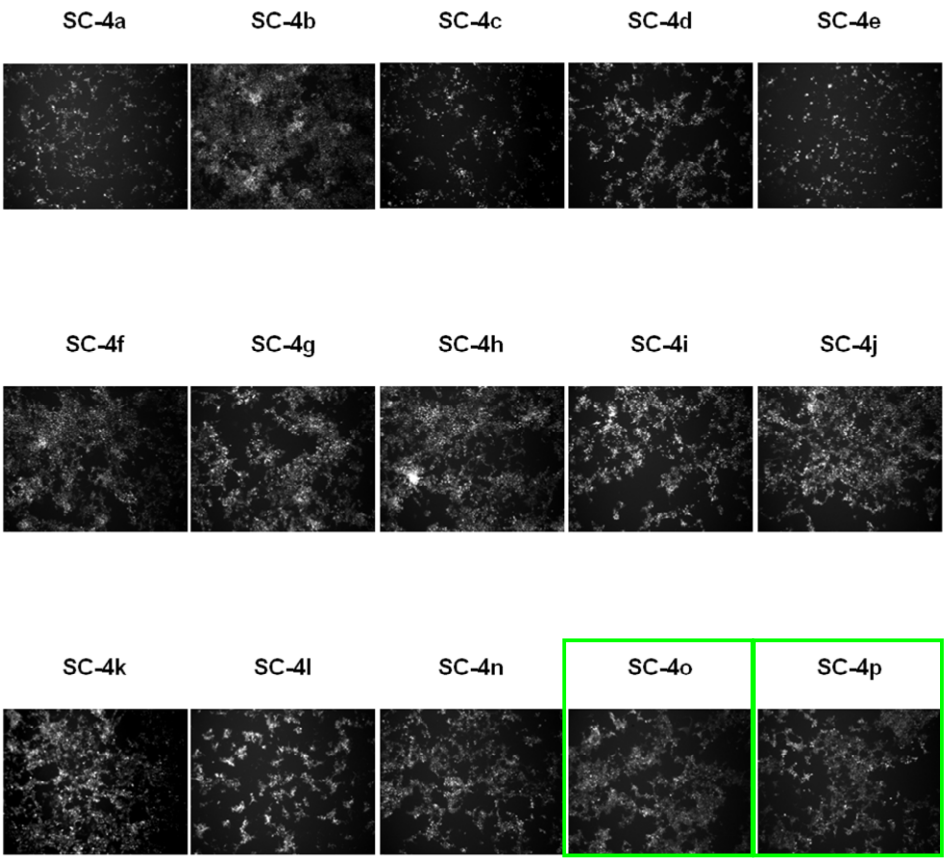

Supplementary Figure S3

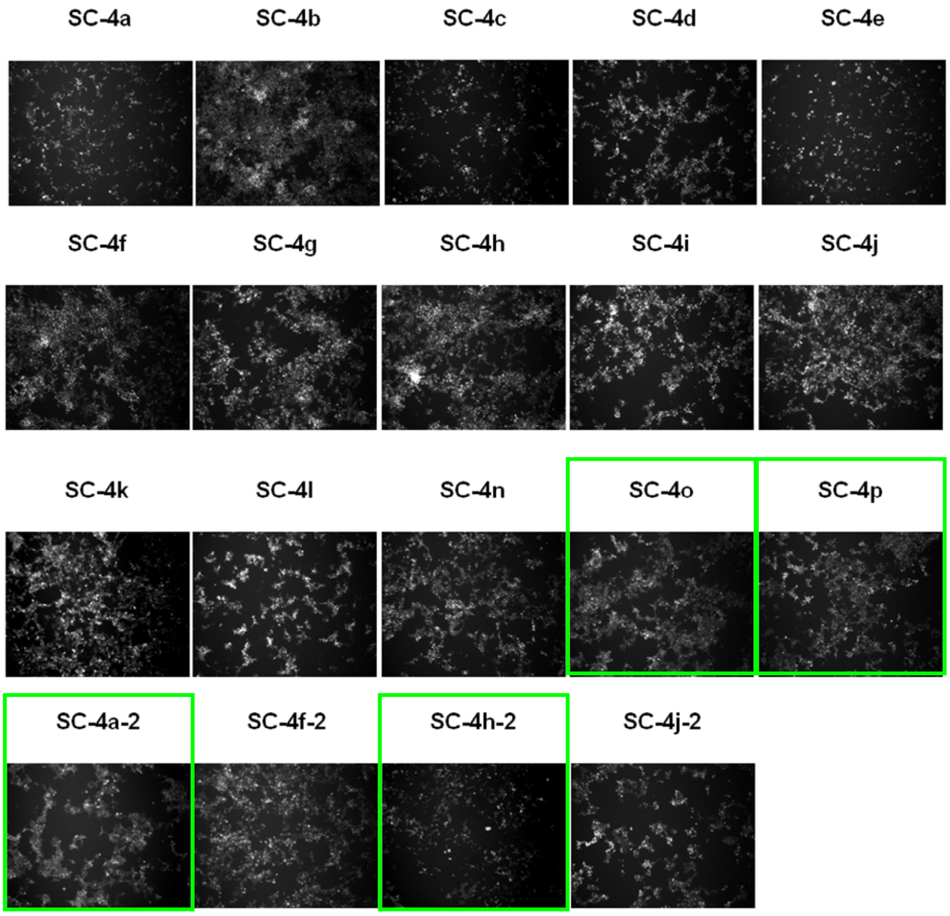

Supplementary Figure S3

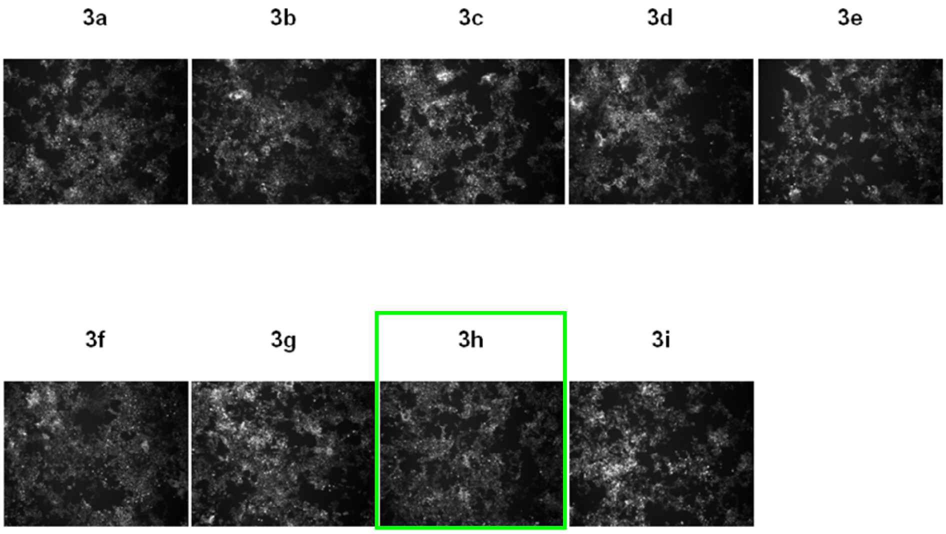

Supplementary Figure S3

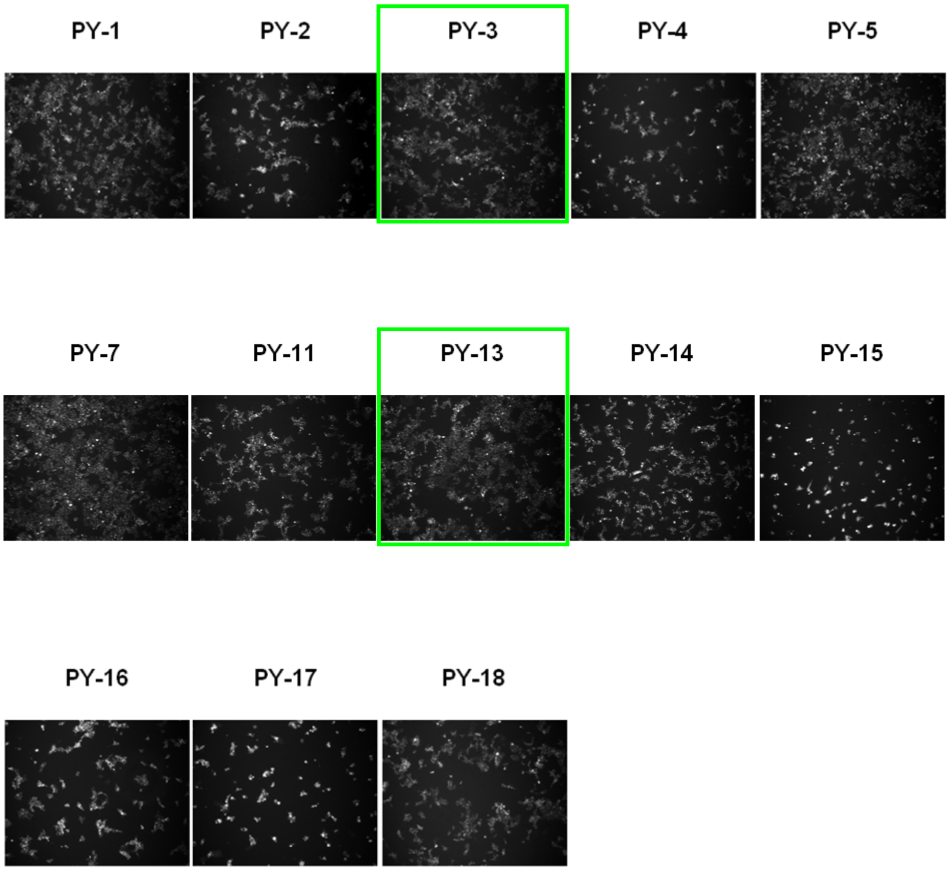

Supplementary Figure S3

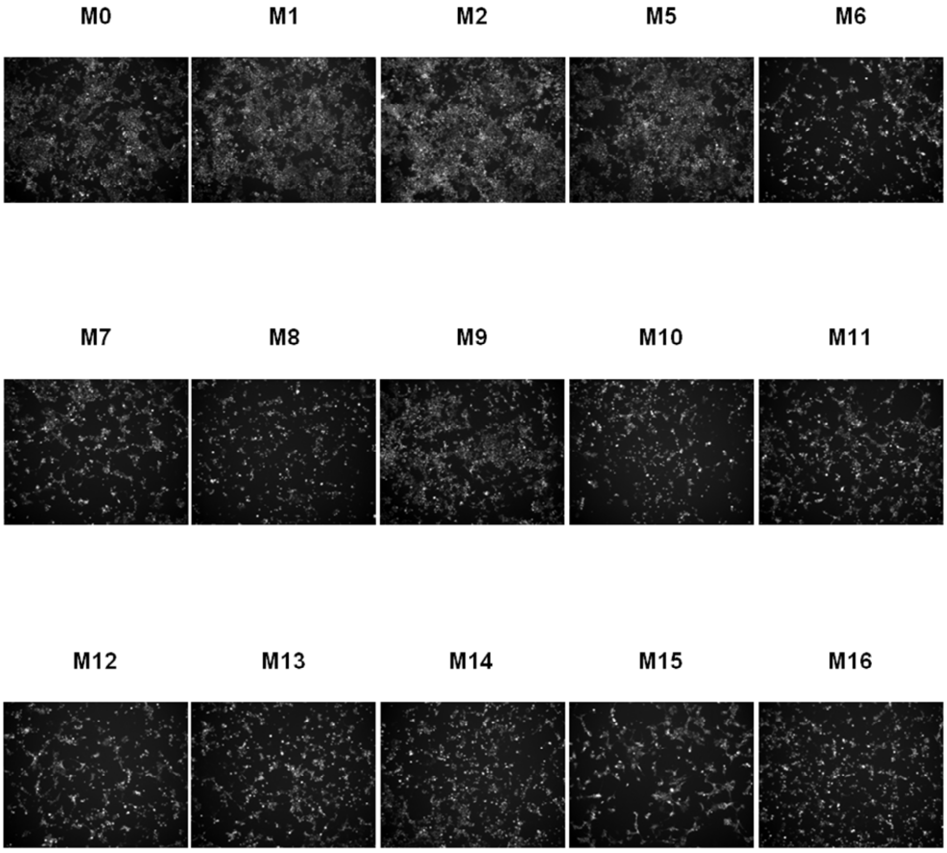

Supplementary Figure S3

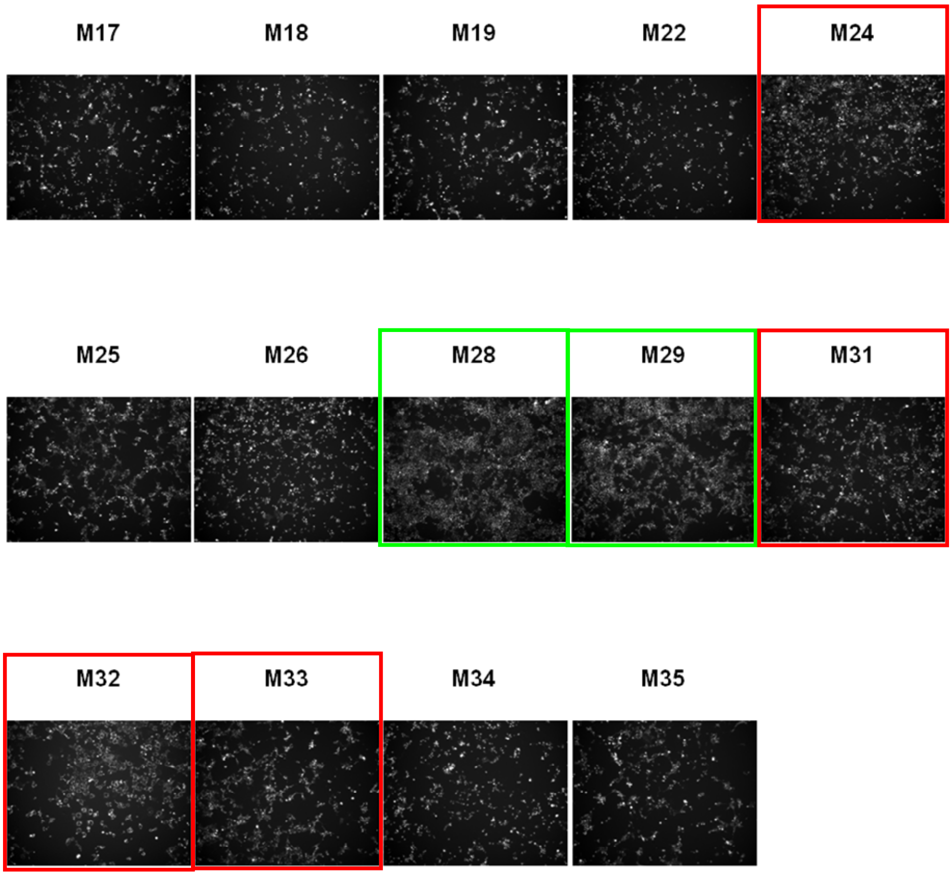

Supplementary Figure S3

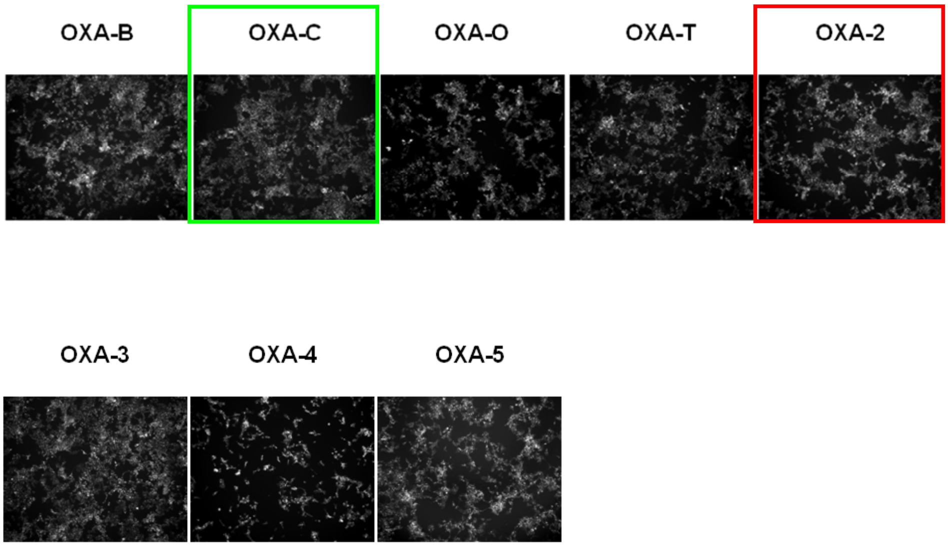

Supplementary Figure S3

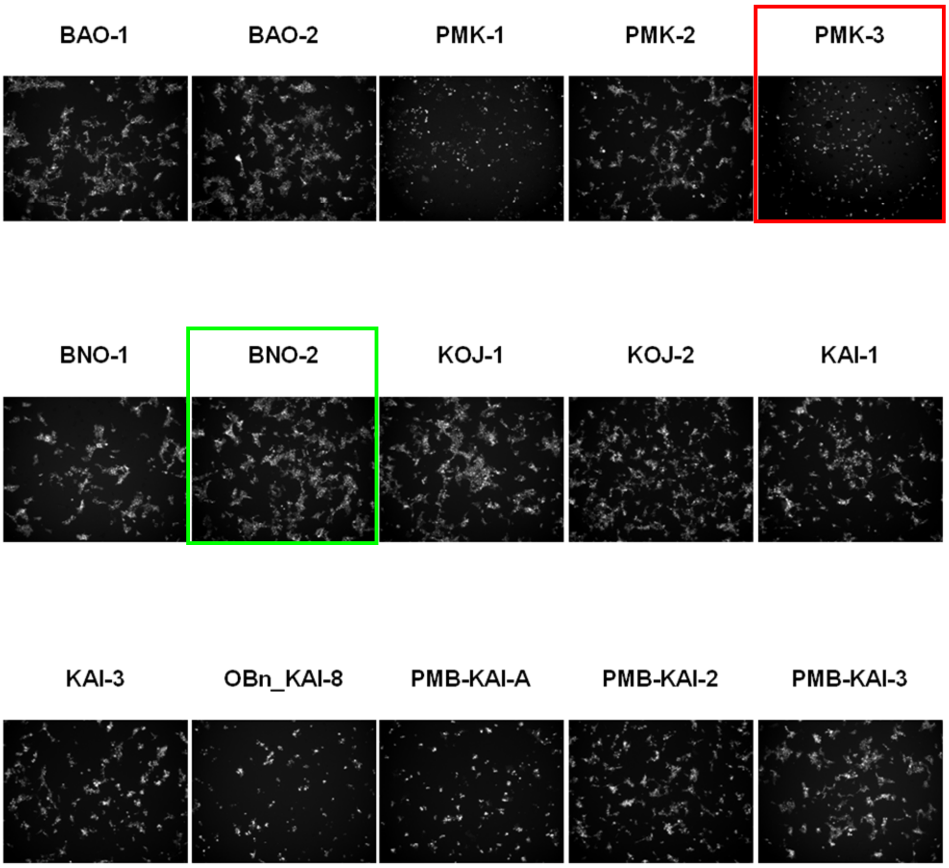

Supplementary Figure S3

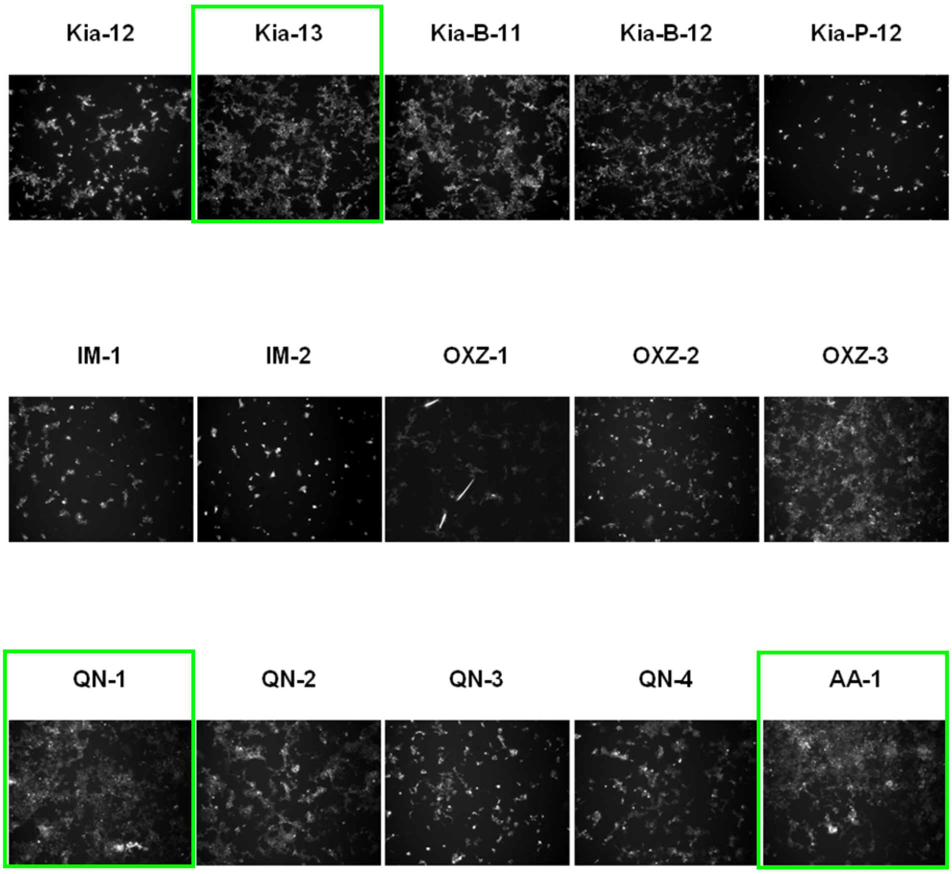

Supplementary Figure S3

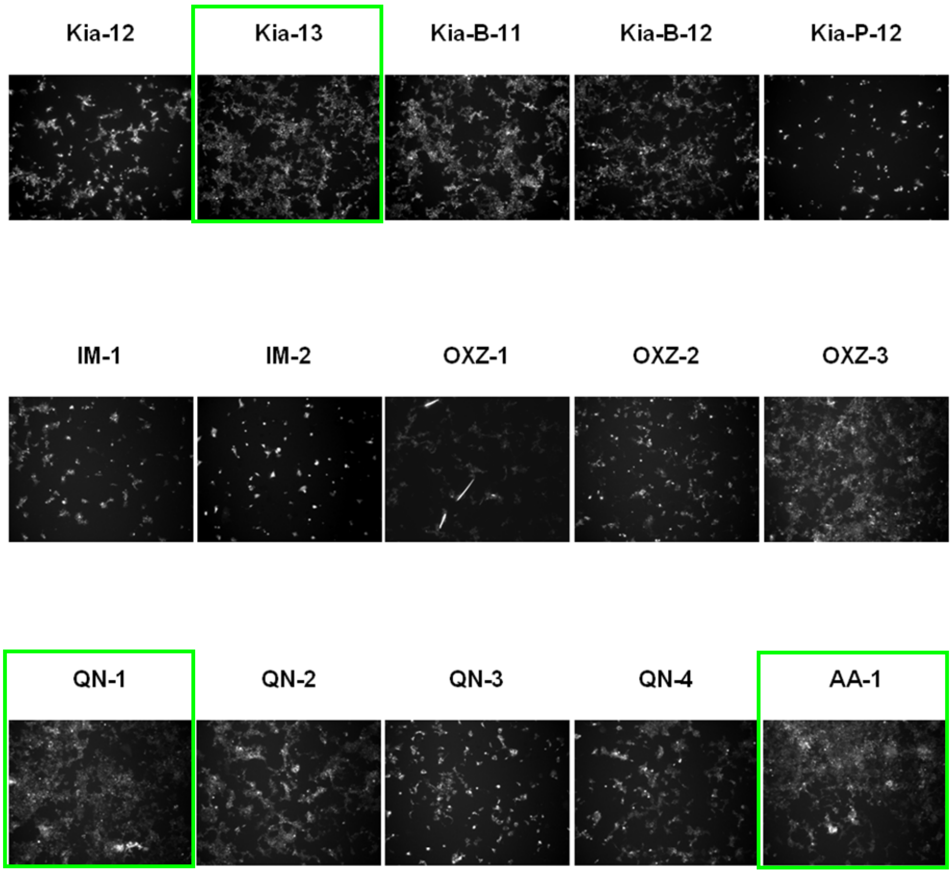

Supplementary Figure S3

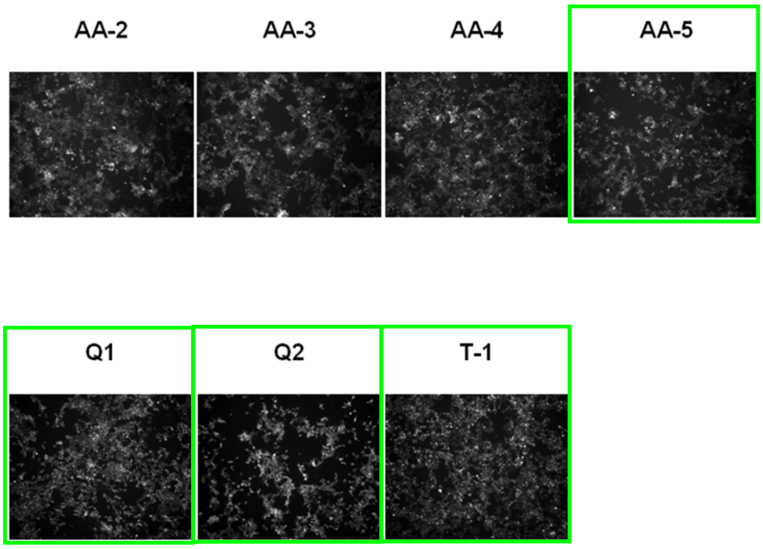

Supplementary Table S1. Primer Sequences

Name	ID	Sequences (5’ → 3’)	Reactions
h_GAPDH_RT_F	NM_002046	CCCCTTCATTGACCTCAACTAGAT	RT-PCR
h_GAPDH_RT_R		CGCTCCTGGAAGATGGTGA	
h_EGFP_F		GTGCTGCTGCCCGACAACCACT	RT-PCR
h_EGFP_R		TCGAGATCTGAGTCCGGACTTGTACA	
R-GAPDH_F_RT1	NM_017008	ACCCAGCCCAGCAAGGATAC	RT-PCR
R-GAPDH_R_RT1		TGGGGTCTGGGATGGAATTG	
R-Trip10_L_RT3	NM_053920	AGGACACCCCCATCTACACTGA	RT-PCR
R-Trip10_R_RT3		TCCATCCATCACCCTTGTCTTC	
h_Col2A1_BR_137	NM_001844	TCTAACAATTATAAACTCCAACCACCAA	qMSP
h_Col2A1_BR_138		GGGAAGATGGGATAGAAGGGAATAT	
h_Trip10_L_M1	NM_004240	AAATTTTTTTATTCGTTTTGTGGTC	qMSP
h_Trip10_R_M1		TAAAATCACCCCGTTCTTACG	
h_GSTP1_MSP1_L	NM_000852	AAGGTTAGGAGTTCGAGATTAGTTC	qMSP
h_GSTP1_MSP1_R		CCCGAATAAATAAAATTATAAATACGT	
R_Col2A1_MSP_F1	NM_012929	AGGTGGGAGTAGGTTTTAGATTTA	qMSP
R_Col2A1_MSP_R1		ATAAAAATAACTTTTCACCAACAC	
R_Trip10_F_M1	NM_053920	GCGTTTAGTTAGGGTTTTCGC	qMSP
R_Trip10_R_M1		ACTCTTACCGCCGAAAACG	

Supplementary Table S2. List of *p* values after F-test for MTT assay.

DMSO	0 µM	1.875 µM	3.75 µM	7.5 µM	15 µM	30 µM
**Proc.**	**0.176**	**0.699**	**0.612**	**0.895**	**0.516**	**0.255**
**5-Aza**	**0.339**	**0.571**	**0.602**	**0.696**	**0.285**	***0.001****
**IM25**	**0.511**	**0.738**	**0.708**	**0.405**	**0.388**	**0.724**
**IM9**	**0.314**	**0.708**	**0.401**	**0.705**	**0.976**	**0.815**
**CI4-1**	**0.875**	**0.754**	**0.676**	**0.257**	**0.375**	**0.756**
**CI5-1**	**0.235**	**0.179**	**0.940**	**0.544**	**0.640**	**0.530**
**4e**	**0.588**	**0.710**	**0.387**	**0.064**	**0.601**	***0.001****
**4o**	**0.194**	**0.696**	**0.075**	**0.264**	**0.465**	***0.000****

* Significant difference.

Supplementary Table S3. List of *p* values after F-test results for ELISA assay.

DMSO	0 µM	1.875 µM	3.75 µM	7.5 µM	15 µM	30 µM
**Proc.**	0.833	0.370	***0.039****	0.245	0.467	***0.018****
**5-Aza**	0.126	0.753	0.339	0.337	***<0.0001****	***<0.0001****
**IM25**	0.239	0.397	***0.003****	***0.011****	***<0.001****	***0.042****
**IM9**	0.053	0.732	0.250	0.899	***0.047****	0.848
**CI4-1**	0.188	0.832	***<0.001****	***<0.0001****	***<0.0001****	***0.038****
**CI5-1**	0.857	0.336	0.597	0.142	***0.030****	***0.005****
**4e**	0.726	***0.016****	***<0.0001****	***<0.0001****	***<0.0001****	***<0.0001****
**4o**	0.599	0.085	***0.014****	0.069	***<0.001****	***0.008****

* Significant difference.
